# Comparison of intra- and inter-host genetic diversity in rabies virus during experimental cross-species transmission

**DOI:** 10.1371/journal.ppat.1007799

**Published:** 2019-06-20

**Authors:** Emilie M. Bonnaud, Cécile Troupin, Laurent Dacheux, Edward C. Holmes, Elodie Monchatre-Leroy, Marion Tanguy, Christiane Bouchier, Florence Cliquet, Jacques Barrat, Hervé Bourhy

**Affiliations:** 1 Institut Pasteur, Unit Lyssavirus Dynamics and Host Adaptation, WHO Collaborating Centre for Reference and Research on Rabies, Paris, France; 2 Marie Bashir Institute for Infectious Diseases and Biosecurity, Charles Perkins Centre, School of Life and Environmental Sciences and Sydney Medical School, The University of Sydney, Sydney, Australia; 3 ANSES, Nancy Laboratory for Rabies and Wildlife, WHO Collaborating Centre for Research and Management in Zoonoses Control, OIE reference laboratory for rabies, European Union Reference Laboratory for rabies, European Union Reference Laboratory for rabies ser3ology, Malzéville, France; 4 Institut Pasteur, Genomics Platform, Paris, France; University of Michigan, UNITED STATES

## Abstract

The development of high-throughput genome sequencing enables accurate measurements of levels of sub-consensus intra-host virus genetic diversity and analysis of the role played by natural selection during cross-species transmission. We analysed the natural and experimental evolution of rabies virus (RABV), an important example of a virus that is able to make multiple host jumps. In particular, we (i) analyzed RABV evolution during experimental host switching with the goal of identifying possible genetic markers of host adaptation, (ii) compared the mutational changes observed during passage with those observed *in natura*, and (iii) determined whether the colonization of new hosts or tissues requires adaptive evolution in the virus. To address these aims, animal infection models (dog and fox) and primary cell culture models (embryo brain cells of dog and fox) were developed and viral variation was studied in detail through deep genome sequencing. Our analysis revealed a strong unidirectional host evolutionary effect, as dog-adapted rabies virus was able to replicate in fox and fox cells relatively easily, while dogs or neuronal dog cells were not easily susceptible to fox adapted-RABV. This suggests that dog RABV may be able to adapt to some hosts more easily than other host variants, or that when RABV switched from dogs to red foxes it lost its ability to adapt easily to other species. Although no difference in patterns of mutation variation between different host organs was observed, mutations were common following both *in vitro* and *in vivo* passage. However, only a small number of these mutations also appeared *in natura*, suggesting that adaptation during successful cross-species virus transmission is a complex, multifactorial evolutionary process.

## Author summary

Understanding the mechanisms that underpin the cross-species transmission and host adaptation of rabies virus (RABV) remains an important part of the ongoing goal to reduce and eliminate rabies. We utilized next-generation sequencing to perform a deep comparative analysis of the genomic evolution of RABV subpopulations during host adaptation in culture and in animals, with the aim of determining the molecular mechanisms involved in the host-species or tissue adaptation of rabies virus. In particular, we aimed to determine whether experimental evolution can recapitulate evolution in nature. Our results suggest that a limited number of mutations that appeared following both *in vitro* and *in vivo* passage were observed *in natura*. This study also suggests that dog RABV may be able to adapt to some hosts more easily than other host variants.

## Introduction

RNA viruses are characterized by high rates of evolutionary change, the result of replication with error-prone RNA-dependent RNA polymerases and a combination of natural selection and sometimes recombination, enabling rapid adaptation to changing environments, including new host species and cell types [1, 2]. Understanding the evolutionary mechanisms that shape virus genetic diversity is therefore central to determining cross-species transmission and potentially disease emergence.

Rhabdoviruses are RNA viruses that experience frequent cross-species transmission [3]. A good example is provided by rabies virus (RABV) (genus *Lyssavirus*), which constitutes an informative model to explore the evolutionary processes involved in the cross-species transmission and adaptation of RNA viruses to new hosts [4, 5]. RABV, a zoonotic negative single-strand RNA virus, is the main etiological agent of rabies that is still responsible for ~59,000 human deaths annually, mostly in low income countries [6]. The RABV genome encodes five proteins: the nucleoprotein (N), the phosphoprotein (P), the matrix protein (M), the glycoprotein (G) and the polymerase (L). RABV has a worldwide distribution in a wide range of mammalian hosts, especially within the orders *Carnivora* and *Chiroptera*. Phylogenetically, two major groups of RABV have been described, the bat- and dog-related RABVs, each further subdivided into several geographical clades [7]. RABV has also been identified in many mammals, including dogs, bats, skunks, foxes, mongooses, ferret-badgers, and raccoons [8, 9], although in both Asia and Africa domestic dogs are the main reservoir and vector for RABV transmission (including to humans).

To date, the evolution of RABV in a new environment has largely been studied through the use of consensus sequences of virus genomes. In contrast, little is known about the relationship between RABV intra-host diversity, including subpopulation differentiation in different hosts and tissues, and the patterns and processes of evolutionary change observed *in natura*. Although several studies have investigated the nature of RABV genetic diversity following adaptation to cell lines [10, 11], to experimental hosts [10, 12–14], or in the context of vaccines [15–17], to date no strong host-specific molecular fingerprints have been identified using either Sanger [18] or next-generation sequencing (NGS) [7, 19].

The cross-species transmission of viruses, and of RABV in particular, results from a complex combination of eco-epidemiological factors and genetic factors conditioning the specific virus-host relationship [4, 9]. Epidemiological factors, such as the probability of efficient contact (i.e. the frequency of contacts and inherent characteristics related to the type of contact favoring or not the transmission), animal population densities, human population densities, niche overlap, as well as the phylogenetic relatedness between reservoir and the new host are of fundamental importance in determining the probability with which a virus can jump from one animal species to the other and develop sustained transmission cycles [3, 20, 21]. However, ecological factors alone are likely insufficient to explain the capacity of a virus to emerge in a new species, such that genetic changes that facilitate host adaptation are needed to increase virus fitness in the new host environment. Therefore, understanding the mechanisms of host adaptation and interspecies transmission of RABV remains an important part of the ongoing goal to reduce and eliminate rabies.

The aim of this study was to obtain new insights into the molecular mechanisms of RABV host species or tissue adaptation and hence to reveal the impact of distinct environments on virus evolution. To this end we took advantage of NGS technologies to perform a comparative analysis of the deep genomic diversity and evolution of RABV subpopulations sampled during host adaptation. As a model system, we used the well characterized RABV cross species transmission from the domestic dog (*Canis lupus familiaris*) to the European red fox (*Vulpes vulpes*) [22, 23]. The dog RABV used in this study belongs to the Cosmopolitan lineage from which the fox-adapted RABV emerged in Europe [22, 23]. Serial intra- and inter-host experimental passages both in animals (dogs and foxes) and in primary brain cell cultures from dog and fox embryo origin were performed, followed by NGS to dissect RABV microevolution and reveal the pattern of virus evolution and adaptation. Importantly, these inter-host passages aim to experimentally mimic the process of virus adaptation to a new host species and determine if this selective process leads to the appearance of new dominant viral genotypes. In animals, neurotropic RABV has to replicate in various organs other than brain, from muscle at the site of inoculation to salivary glands at the late stage of infection [8]. Therefore, to determine if replication in different cell types involved active adaptation, we also analyzed viral subpopulations sampled from different organs of each infected animal [24, 25]. Finally, we asked whether the same mutations that have occurred during microevolution in the passage experiments performed here mirror those that have occurred in various RABV hosts sampled *in natura* [7].

## Results

### Fox adapted-RABV encounters difficulties when infecting dogs

**Fig 1:**
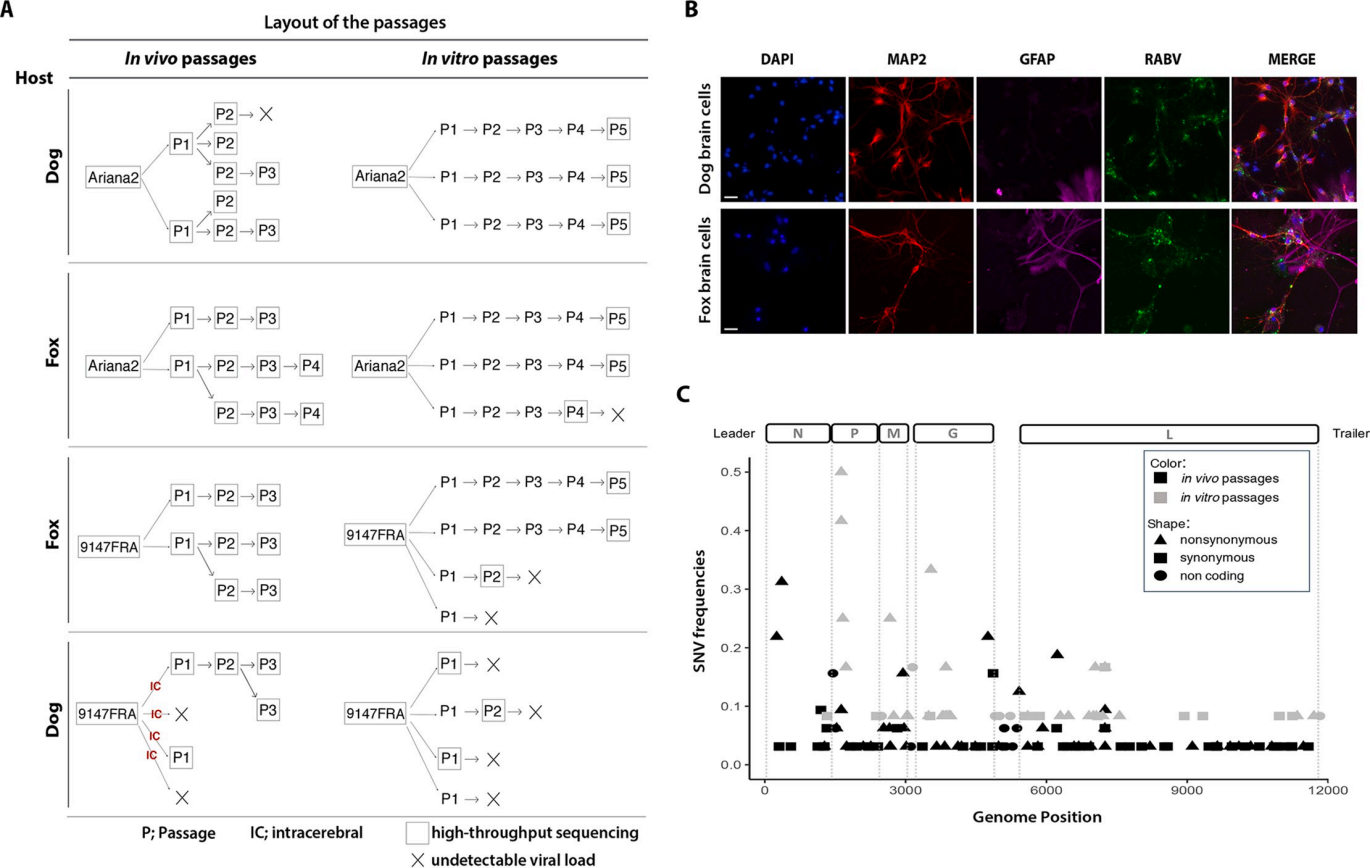
Genetic variability after experimental passages of RABV *in vitro* and *in vivo*. (A) Layout of the passage experiments. For the *in vivo* experiment, RABV was inoculated by intramuscular injection, with the exception of the first ‘vFox on Dog’ passages that were performed by intracerebral inoculation (IC). Viruses were isolated on each animal both from salivary gland and brain, titrated by qPCR and by intracerebral inoculation and sequenced. Virus isolated from salivary gland was used as inoculum for the subsequent animal passage. For the *in vitro* experiment, all passages were titrated to control the infectivity and only the last one was sequenced. The black cross illustrates passages that have been stopped because of the inability to obtain a enough high viral titer. (B) Images of dog and fox primary brain cell cultures infected with RABV (vfox 9147FRA and vdog Ariana2). Neuronal cells and astrocytes were stained with anti-MAP2 and anti-GFAP antibodies, respectively. Images were obtained using a Zeiss Axioplan fluorescence microscope equipped with a Zeiss ApoTome system (obj.10X). Bars = 50μm. (C) RABV intrinsic variability characterized by high-throughput sequencing. A schematic representation of RABV genome is shown at the top of the figure. ORFs are delimited by dotted grey lines. Frequencies of RABV Single Nucleotide Variations (SNVs), after *in vivo* (in black) or *in vitro* (in gray) experimental passages, are graphically represented along the genome. Different types of SNVs detected; nonsynonymous, synonymous or non-coding, are characterized by different shapes. An arbitrary cut-off frequency of 2% was used to validate variants as significantly different from artefactually introduced reverse-transcription, PCR and sequencing errors. Abbreviations: vDog—Ariana2 strain; vFox - 9147FRA strain.

To help determine the molecular mechanisms associated with RABV adaptation to a new host species, we developed two experimental passage models. Specifically, intra- and inter-host passages of dog or red fox adapted-RABV (vDog and vFox, respectively) were performed both *in vivo* in dogs and red foxes, and *in vitro* on primary brain cell cultures from dog and red fox embryos. The layout of the different passages is illustrated in Fig 1A. Primary brain cell cultures were successfully infected by RABV strains (Fig 1B). However, transmission of fox adapted-RABV on dog host (‘vFox on Dog’) was unsuccessful (Fig 1A), while the other heterologous and homologous passages were more easily achieved. Specifically, two attempts at the ‘vFox on Dog’ transfer using the intramuscular route of infection failed and no trace of virus was detected either in the brain or in the salivary glands of dogs by RT-qPCR. Then, in an attempt to facilitate transmission of the virus, infections by the intracerebral route were performed. In this case, only 2 of the 4 trials gave positive results and led to the infection followed by a rapid death of the animals (Fig 1A, last panel on the left). The same difficulties were faced when performing the heterologous ‘vFox on Dog’ passages *in vitro*. Passages could not be performed further than passages P1 or P2 because of a rapid loss of virus detection, indicating a marked incapacity of vFox to grow on dog cells and be serially transmitted. Hence, dogs or dog embryo brain cells do not seem to be susceptible to fox adapted-RABV compared to foxes or fox embryo brain cells. In this regard, and as expected, our data also suggest that dogs infected by the intracerebral route were more susceptible than those infected by the intramuscular route.

### Viral genetic diversity after *in vitro* and *in vivo* RABV experimental passages

**Fig 2:**
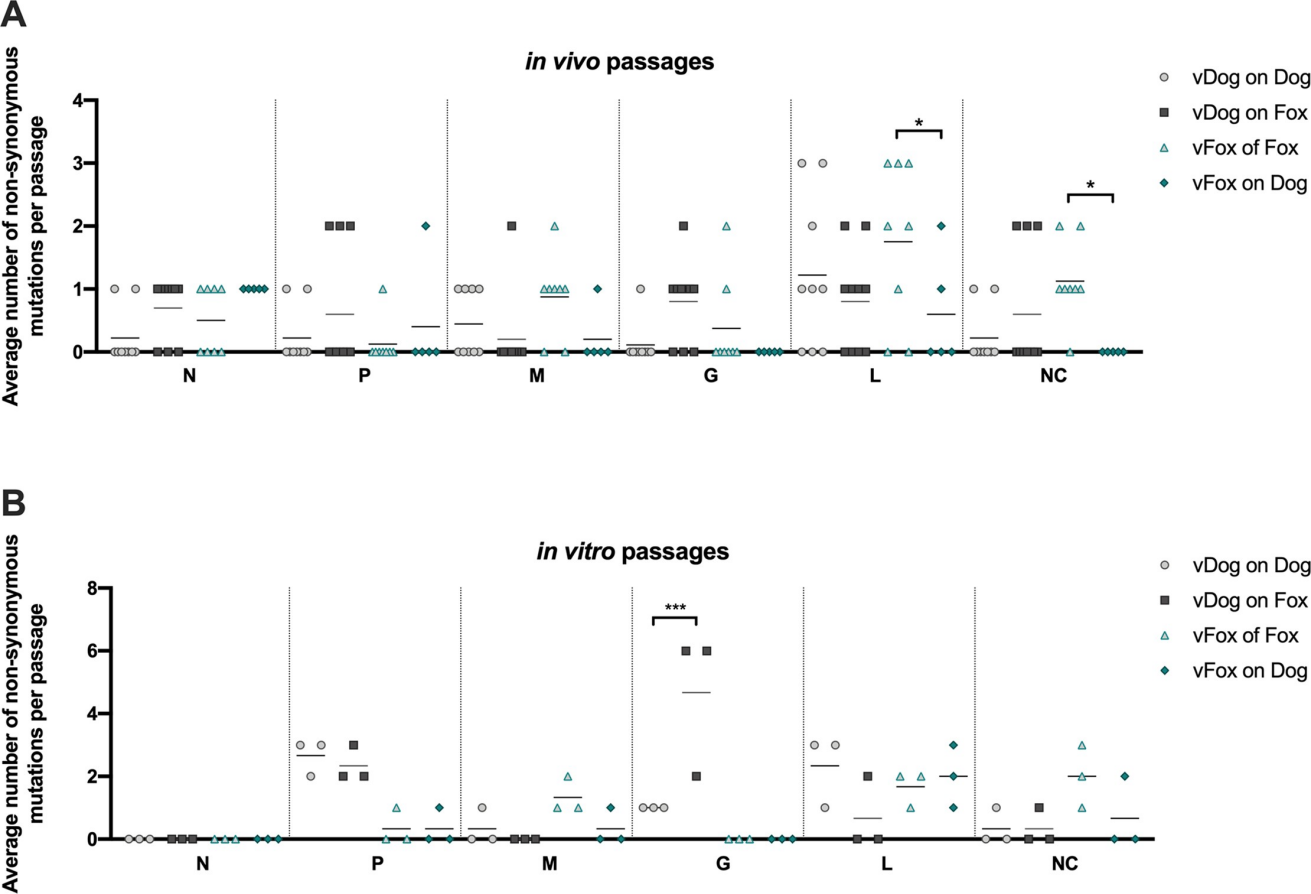
Distribution of non-synonymous mutations across the RABV genome. The number of non-synonymous mutations were examined in each region of RABV genome (N, P, M, G and L genes and the non-coding (NC) regions) and are represented by a dot plot. The average number of mutations were assessed per region and per animal for *in vivo* passages (A) or per cell culture for *in vitro* passages (B). Statistical analysis (Tukey’s multiple comparison test) was performed using GraphPad Prism software. * *P* < 0.01; *** *P* < 0.0001. Error bars correspond to standard deviations.

**Fig 3:**
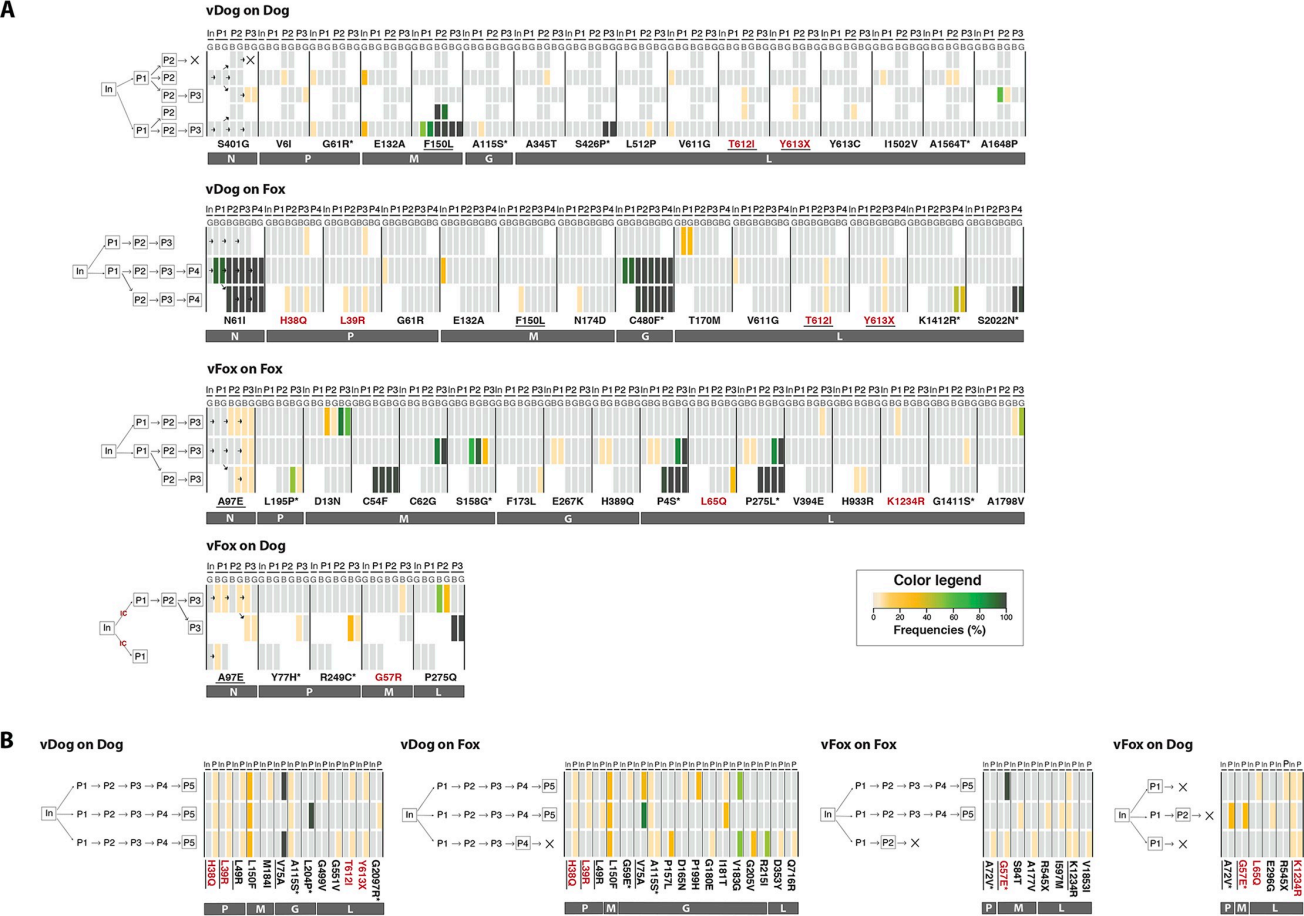
Heatmap of non-synonymous mutations during experimental passage. Nucleotide sites for which variability is detected above a frequency of 2% of the total viral population are shown: (A) *in vivo* and (B) *in vitro* experiments. The frequency of non-synonymous mutations in each passage (P1 to P3) is color coded (see legend). A schematic representation of experiment design is showed on the left part of each heatmap, for details see Fig 1. Mutations highlighted in red are those found both during *in vivo* and *in vitro* experiments. Mutations underlined are those observed both during intra- and inter-host passages, excluding those already observed in the *inoculum*. Mutations associated with an asterisk are those that have also been observed in *in natura* RABV strains [7]. *In*, *Inoculum*; P, Passage; G, Salivary Gland; B, Brain.

At each passage, RABV was analyzed by NGS to characterize genetic diversity. The frequency of all the mutations observed was obtained by comparing the nucleotide sequence of the different samples collected with that of the respective challenge virus (vDog or vFox). The average coverage of RABV genome reached 21,300 and 40,000 reads for *in vitro* and *in vivo* experiments, respectively, and hence is sufficient to allow quantification of minor frequency variants in the viral populations (S1 Fig). To avoid potential artifacts introduced by our amplification and sequencing strategy and to follow a conservative strategy, an arbitrary threshold of frequency of Single Nucleotide Variations (SNVs) present in at least 2% of the total number of reads was further considered for the analysis. Accordingly, 148 SNVs were detected and distributed throughout the genome at both the consensus and sub-consensus levels (Fig 1C). SNVs were located in the five coding regions and in non-coding parts of the genome, with the exception of the N gene coding region of isolates obtained during *in vitro* passage (Fig 2 and S2), and the number of substitutions does not significantly vary according to the number of passages (S3 Fig). However, and in contrast to what was previously observed at the consensus level *in natura* [7], fewer mutations were seen in the non-coding compared to the coding regions. Of these 148 SNVs, 7 were observed in both the *in vitro* and *in vivo* experiments (Fig 3).

The total number of SNVs at both the consensus and sub-consensus level per passage (i.e. frequency of SNVs) was slightly higher during *in vitro* than *in vivo* passage (Fig 1C, Table 1): the numbers of all SNVs relative to the number of samples observed during *in vivo* or *in vitro* experiments were 21 and 28, respectively (Table 1). The percentage of non-synonymous mutations was also higher *in vitro* than *in vivo* (71% versus 53%, respectively), especially in the fox (Table 1). Additionally, the number of dominant SNVs–that is, those present in more than 50% of reads–was four times higher in animals than in cells, while the opposite was observed for the introduction of premature stop codons (frequencies of 0.4 and 1.7 in animals and in cells, respectively) in the different viral gene sequences (Table 1). All of these parameters indicate that RABV genetic diversity is greater *in vitro* than *in vivo* passage.

**Table 1:** Analysis of RABV genetic variation in intra- and inter-host comparisons and in specific organs.

Type of experiment	No. of samples	No. total SNVs (10^−4^ sub./site/ passage)^*a*^	No. Non-Synonymous SNVs	No. dominant SNVs ^*b*^ (10^−4^ sub./site/ passage)^*a*^	No. deleterious SNVs	Ratio of No. of SNVs in the brain / No. of SNVs in salivary gland (Br/SG)	Br/SG for dominant SNVs
***In vivo passages experiment***							
**vDog on Dog**	9	4.3 (3.66)	2.1	1 (0.08)	0.2	1.1	1
**vDog on Fox**	10	5.5 (4.65)	3.1	1.9 (1.60)	0.2	1.5	1
**vFox on Fox**	8	7 (5.92)	3.8	2.9 (2.45)	0	1.1	1
**vFox on Dog**	5	4.2 (3.55)	2.2	0.6 (0.05)	0	1.5	1
**Total**	32	21 (4.45)	11.2	6.4 (1.05)	0.4	N/A	N/A
***In vitro passages experiment***							
**vDog on Dog**	3	7.3 (6.20)	6.3	1 (0.08)	0.7	N/A	N/A
**vDog on Fox**	3	9.7 (8.17)	7.7	0.3 (0.02)	0	N/A	N/A
**vFox on Fox**	3	6.7 (5.63)	3.3	0.3 (0.02)	0.3	N/A	N/A
**vFox on Dog**	3	4.3 (3.66)	2.7	0	0.7	N/A	N/A
**Total**	12	28 (5.92)	20	1.6 (0.07)	1.7	N/A	N/A

An arbitrary cut-off frequency of 2% was used to validate variants as significantly different from artefactually introduced reverse-transcription, PCR and sequencing errors. All data expressions are relative to number of samples (animal and cell passages analyzed). ^a^Number of substitutions is divided by the total length of the sequenced genomes (11826 nucleotides). ^b^Number of substitutions present in more than 50% of the reads. Abbreviations: N/A Not Applicable.

FOR ALL: data expression are relative to number of samples (animals or sequences for respectively in vivo and in vitro experiments)

a Number of substitutions is divided by the total length of the sequenced genomes (11826 nucleotides)

b Number of substitutions present in above more than 50% of reads N/A: Not Applicable

To quantify the genetic diversity introduced during these experimental passages, we first analyzed all SNVs and calculated the total number of mutations per site and per experimental passage. Accordingly, the value observed *in vitro* (5.92 x 10^−4^ subs/site/passage) is slightly higher than that observed *in vivo* (4.45 x 10^−4^ subs/site/passage) (Table 1). This is in accordance with the higher SNVs frequency observed *in vitro* (Fig 1C). Next, we focused on the dominant SNVs. In this case, the value observed in animals (1.05 x 10^−4^ subs/site/passage) is 15 times higher than in cells (0.07 x 10^−4^ subs/site/passage) indicating that a larger proportion of the SNVs are dominant in animal passages (Table 1).

### Patterns of genetic variation in specific organs

We also compared the SNV diversity between brain and salivary glands, two key tissues for RABV spread and pathogenesis. Several SNVs present only in one of the two organs were detected, but the ratio between brain and salivary glands SNVs (ratio Br/SG) remained close to 1 whatever the type of passage considered (Table 1). In addition, the number of dominant SNVs for each type of passages resulted in a ratio Br/SG equal to 1. Hence, our *in vivo* experimental model revealed no difference in patterns of variations between the different organs studied.

### Patterns of intra- and inter-host genetic variation

To help identify different evolutionary pathways when viruses are passed onto homologous or heterologous hosts, we compared the RABV genetic diversity and the frequency of mutations (related to the respective challenge virus) between intra- and inter-host passages at the genome and individual gene levels. This revealed no significant differences in the total number of mutations on a genome-wide scale (Table 1). The number of mutations observed varies according to the 5 different genes: 3 in the N, 9 in the P, 10 in the M, 15 in the G and 28 in the L gene. In terms of the percentage of the length of each gene, this gives the following ascending order 0.2% for the N gene, 0.4% for the L gene, 1% for the P and G genes, and 1.6% for the most variable M gene. However, greater variability seems to be associated with foxes as hosts, both during *in vivo* and *in vitro* experiments: 5.5 and 7 SNVs for ‘vDog on fox’ and ‘vFox on fox’, respectively, compared to 4.3 and 4.2 for ‘vDog on dog’ and ‘vFox on dog’, respectively, during *in vivo* passage; 9.7 and 6.7 for ‘vDog on fox’ and ‘vFox on fox’, respectively, compared to 7.3 and 4.3 for ‘vDog on dog’ and ‘vFox on dog’, respectively, during *in vitro* passage. There is, however, no clear explanation for this observation. To identify any specific evolutionary pathway for individual genes, we analyzed the average number of non-synonymous mutations per passage for each gene. Only small variations in the number of these mutations were observed in *in vitro* passages, even though this number is significantly higher in the L coding region in vFox on fox than in vFox on dog (Fig 2B). More interestingly, we detected a significant increase in the number of these mutations in the G gene during inter-host ‘vDog on Fox’ passages compared to the corresponding intra-host ‘vDog on Dog’ (Fig 2A).

### The accumulation of non-synonymous mutations during experimental passage

An heatmap was used to analyze the observed rate of mutation during each experimental passage and to assess the genetic variability and evolution of RABV along the passages (Fig 3). This revealed several interesting features: (i) some mutations were observed both during *in vivo* and *in vitro* experiments (Fig 3, red characters: mutations H to Q in position 38 of the P protein (P-H38Q), P-L39R, M-G57R or E, L-L65Q, L-T612I, L-Y613X, L-K1234R); (ii) approximately 50% of these mutations have a frequency among global RABV populations of less than 10% (light yellow rectangles); (iii) in some passages mutations appear in both the brain and the salivary glands and are further transmitted in the following passages, the most significant of which were: M-F150L in ‘vDog on dog’; N-N61I and G-C480F in ‘vDog on fox’; M-C54F, L-P4S and L-P275L in ‘vFox on fox’; (iv) all mutations whose frequency was above 50% were observed both in the brain and in the salivary glands, although this was not always the case for other minor variants; and (v) very few mutations are present in all the replicates (*in vivo*: N-A97E; *in vitro*: P-H38Q, P-L39R).

Some mutations were detected both during intra- and inter-host passages (Fig 3, underlined characters). However, the dominant mutations observed during intra-host passages were never found in the corresponding inter-host passages (for vDog: M-F150L, L-S426P; for vFox: M-C54F, M-C62G, L-P4S, L-P275L). Furthermore, we observed more mutations in foxes as hosts, and particularly more high frequency SNVs (Table 1).

An analysis of the appearance of single mutations throughout passages revealed that most remained at low levels (Fig 3). However, in some cases mutations appeared spontaneously as dominant mutations during the first passages and rose to high frequency in the last passage(s), notably the *in vivo* mutations N-N61I, M-D13N, M-C54F, M-F150L, G-C480F, L-P4S, L-P275L, L-P275Q. In contrast, other mutations dropped in frequency during the passages (*in vivo*: N-A97E, M-S158G, L-P275L, L-P275Q), while others appeared in independent passages (*in vivo*: L-T612I, L-613X, P-H38Q, P-L39R, N-A97E; *in vitro*: G-V75A, L-T612I, L-613X, P-H38Q, P-L39R, G-I181T, G-V183G, M-G57E, L-K1234R).

### Mutations that appear in experimental passages are present *in natura*

To examine if the mutations found in experimental passages are also present *in natura*, we took advantage of a previous and well documented phylogenetic study of RABV performed on a genome-wide and global scale [7]. From these data we limited our analysis to the 254 sequences associated with RABV in non-flying species. Interestingly, 19 of the 65 different mutations (~30%) detected during passages were also found *in natura* (Fig 3, asterisk). Among these 19, only 7 were dominant mutations: M-G57E, M-S158G, G-L204P, L-P4S, L-P275L, L-S426P, L-2022N.

**Fig 4:**
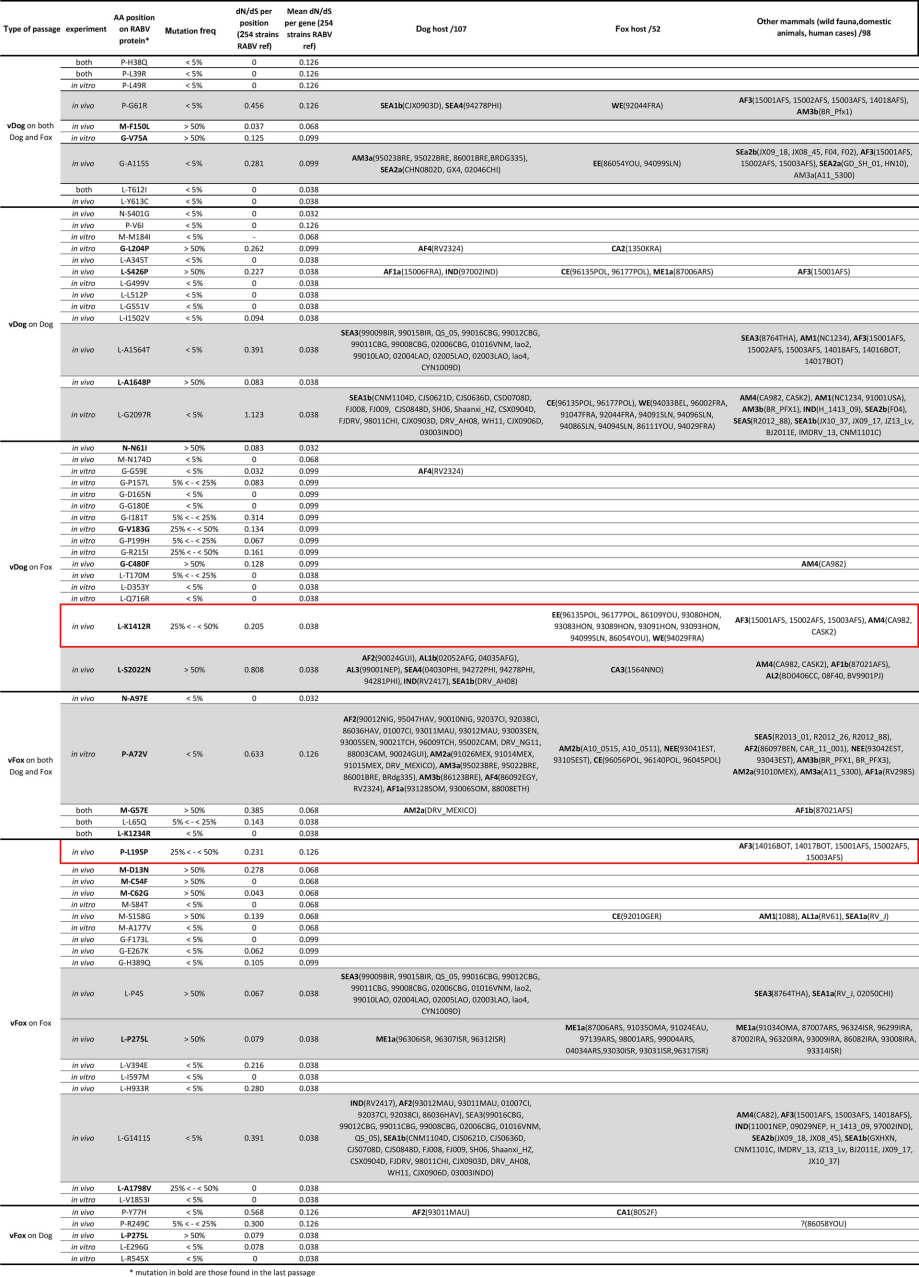
Comparison of mutations observed *in vitro* and *in vivo*. Description of non-synonymous mutations observed during *in vitro* and *in vivo* experiments. For the list of isolates in which the same mutations was observed *in natura*, the name of the lineage is indicated in bold according to the previous defined nomenclature [7]. Mutations highlighted in red boxes are those observed during experimental passages in foxes but never in dogs or in dog-RABV *in natura*. Mutations in bold are those observed in the last passage of each type of passages. Mutations highlighted in grey are observed in a large set of sequences observed in the 3 categories of host species. The following code has been used to describe the mutations: P-H38Q denotes a mutation from H to Q in position 38 of the phosphoprote.

To determine whether a mutation that appears and is maintained during passages (i.e. dominant mutation observed until last passages; mutations indicated in bold in Fig 4) may be subject to adaptive evolution in nature, we subtracted the value obtained for the ratio of non-synonymous (*d*_*N*_) to synonymous (*d*_*S*_) substitutions per site with that of the mean *d*_*N*_/*d*_*S*_ for each gene involved, and defined it as the relative *d*_*N*_*/d*_*S*_ (i.e. r*d*_*N*_*/d*_*S*_) (Fig 4). We used this approach because of the very short time-scale of passage evolution that necessarily means high frequency non-synonymous changes might rather reflect transient deleterious mutations than selectively advantageous ones [26]. For mutations M-C54F (‘vFox on fox’), M-C62G (‘vFox on fox’), M-F150L (vDog on both dog and fox), L-K1234R (vFox on both dog and fox) and L-A1798V (‘vFox on fox’), the r*d*_*N*_*/d*_*S*_ is negative, indicating that these sites are under strong purifying selection. However, all other dominant mutations are characterized by a positive r*d*_*N*_*/d*_*S*._ Therefore, these latter positions are evolving neutrally or are subject to positive selection. Some of the mutations observed in our experimental passages were not found in the *in natura* sequences or only sporadically (i.e. P-Y77H, P-R249C, M-G57E, M-S158G, G-G59E, G-L204P, G-C480F, L-426P) and so could not be clearly attributed to host adaptation. We looked in more detail at those mutations that appeared in experimental passages that were also found *in natura*, particularly to distinguish those that might be attributed to RABV adaptation to different species of naturally infected hosts (dog, fox or other mammals) [7] (Fig 4). As mentioned before, 2% is taken as an arbitrary threshold but we also provided results for nonsynonymous mutations at other thresholds (2, 5, 25 and 50%; see Fig 4) indicating that our arbitrary cut-off of 2% had no major impact on the conclusions drawn. Nine mutations (P-G61R, P-A72V, G-A115S, L-P4S, L-P275L, L-G1411S, L-A1564T, L-2097R, L-2022N) were observed in the three categories of host species (Fig 4, rows highlighted in grey), suggesting that these sites could be highly variable RABV positions not subject to any functional or structural constraints. Conversely, three other mutations could have played a role in the adaptation process (Fig 4). The dominant mutation L-P275L detected during *in vivo* ‘vFox on Fox’ passages was exclusively observed among RABV of the ME1a clade (viruses circulating in the Middle East), a phylogenetic group predominantly composed of fox and wildlife animals [7]: this could in theory represent a molecular signature of fox-related RABV strain. In the same experimental passages, the mutation P-L195P is never found in dog-adapted RABV, but only in an Africa 3 clade: a phylogenetically distinct monophyletic group composed of RABV circulating in Southern Africa and adapted to mongoose [27]. More interestingly, mutation L-K1412R detected during *in vivo* ‘vDog on Fox’ passage is never observed in dogs, but mostly found in foxes and in a few mongooses and skunks. As such, this could represent a molecular signature of the adaption of dog-related RABV to wildlife animal species, and the fox in particular.

## Discussion

Cross-species virus transmission leading to disease emergence is a major public health concern.

When viruses first contact a novel host, they are often poorly adapted, either because they replicate poorly or are rapidly eliminated by the host immune response, and therefore fail to either propagate in the body of the infected host or establish onward transmission in the new host population. This evolutionary process is clearly illustrated in the case of RABV, in which most host jumps result in only transient spill-over infections, with relatively few establishing sustained transmission cycles in the new host [5, 7, 8, 18]. Therefore, RABV, a rapidly evolving virus that transmits to multiple hosts [10, 12, 14], is an interesting model to explore the early evolutionary mechanisms involved in adaptation to a heterologous host [7]. However, the identification of such adaptive genetic changes is often difficult [25]. Although virus transmission between different host species based on viral gene sequences has commonly been studied *in natura*, to date these studies have been unable to identify either consensus level mutations that are clearly subject to positive selection or specific genetic signatures associated with host switching [4, 7, 13, 28, 29].

In an attempt to mimic the transmission of the dog-adapted rabies virus to foxes that happened in Europe in the early 19^th^ century, we analyzed the evolution of RABV during experimental passages performed *in vivo* and *in vitro* [7, 22]. Our results revealed a clear host-specific pattern of virus genetic variation. Indeed, the difficulties we faced in both the *in vitro* or *in vivo* transmission of fox RABV into dogs or dog primary embryo brain cells, compared to the capacity of the dog RABV to more easily infect foxes, illustrate the fundamental role that genetic background plays in the adaptive process. This is in accordance with the observed capacity of RABV to jump species boundaries in nature, in which the dog-adapted rabies virus has shifted to fox rabies-adapted viruses independently in many parts of the world, but where the reverse fox-to-dog transfer has never been observed to date [7, 19, 22, 23]. It is also in agreement with former studies demonstrating, experimentally, the generally high susceptibility of foxes to dog-adapted RABV [30, 31]. The potential role for host phylogeny to constrain the diversity of RABV reservoirs has received considerable attention [5, 32]. However, our study reveals that this relationship is unidirectional for unknown reasons, as the experimental passages from dog to fox does not seem to have the same probability that from fox to dog.

As genetic heterogeneity, adaptive capacity, and evolutionary rate are dependent on an interconnected set of parameters, we attempted to control the virus population size used for each passage as much as practically possible and maintain it at a sufficiently high level to avoid the transmission of small numbers of infectious particles (i.e. a population bottleneck) which would have greatly impacted our observations. Further, at least three independent passages were obtained for each type of *in vitro* and *in vivo* passage (with the exception of fox RABV in dogs was unsuccessful) and heterologous passages were always performed in parallel to the same set of experiments run in homologous conditions.

In contrast to previous studies, the deep full genome sequencing approach used here allowed us to combine thorough evolutionary studies performed at the level of the consensus sequence with a comprehensive analysis of mutations at the sub-consensus level. Using these experimental settings, we observed clear variation in the extent of genetic diversity, SNV frequency, and the percentage of non-synonymous substitutions according to passage type. In particular, the mutational frequency per passage observed at the consensus level in animal passages (1.05 x 10^−4^ mutations/site/passage) was 15 times greater than in cells (0.07 x 10^−4^ mutations/site/passage). Importantly, however, these rates cannot be directly compared to the nucleotide substitution rate observed *in natura* [7, 19, 33], as the latter depicts genetic diversity following the action of natural (especially purifying) selection, whereas purifying selection will have less time to act during experimental passage. It is also tempting to speculate that this difference could indicate that one animal passage corresponds in average of about 15 cell passages, although this may be too simplistic and clearly merits further study. Finally, no difference in patterns of genetic variation was observed between the different organs in infected hosts, suggesting that RABV has not evolved organ-specific differences during propagation from the brain to extra-neuronal organs such as the salivary glands via sensory innervations.

The number of mutations observed varied among the 5 different genes in the ascending order N, L, G, P, and M, which is close to that observed in natural evolution except for the high diversity observed in the M protein [7]. Also of note was that more mutations were observed in the G gene in *in vitro* passages, as previously observed [10, 18, 34]. This is not surprising as the G protein is particularly important from the adaptive perspective, as it is responsible for recognition of host cell receptors and membrane fusion, promotes virus dissemination between infected cells, and is involved in the stimulation of host immune responses [8].

More striking was that the genetic diversity of dog adapted RABV at the consensus and sub-consensus level was greater during the attempts to infect foxes than dogs (either *in vitro* or *in vivo*). This is concordant with the observation that high evolutionary rates facilitate rapid adaptation to new environments [35], and corroborates a previous observation of greater sub-consensus population heterogeneity during the early phase of the RABV host shift from the dog to the fox population in Turkey [19]. Similarly, our study also confirms that virus genetic diversity is higher in heterologous passages compared to homologous passages [21], at least in the case of the passage of dog adapted virus into foxes.

Many of the high frequency mutations that arose during the experimental passages are not observed *in natura* and were not strongly correlated with host species. Given the infectious dose used to eliminate a strong population bottleneck effect, these changes likely had some short-term benefit in this host such that they are selectively advantageous. However, that they are clearly not advantageous across RABV as a whole implies that they negatively impact some other aspect of virus fitness in nature and are usually removed by purifying selection.

Two sets of two mutations appeared simultaneously and independently in at least two different lineages of *in vivo* passages: N-N61I and G-C480F that were observed concomitantly during passages of dog virus in fox, and L-P4S and L-P275L that were observed during passages of fox virus in fox. These pairs of changes may reflect the action of epistasis. Indeed, one of the most interesting results of this study was the identification of mutations that could have played a role in host adaptation as they were observed both during the experimental passages and *in natura*. Two dominant mutations fell into this category: L-P275L detected during *in vivo* ‘vFox on Fox’ passages and exclusively observed among RABV circulating in the Middle East and known as ME1a clade, a group largely comprising isolates from foxes and wild animals [7] and P-L195P not found in dog-adapted RABV but only in a phylogenetically distinct group of RABV circulating in Southern Africa and adapted to mongoose [27]. Both these mutations could represent molecular characteristics associated with wildlife-related RABV strains. Why these mutations emerged after a few rounds of experimental passage in foxes although they never appeared in Europe, and one is only found in the Middle East (i.e. lineage ME1), is presently unknown. More striking, the dominant mutation, L-K1412R detected during passages of dog virus on fox, is never observed *in natura* in dogs and mostly found in foxes in two European fox lineages (denoted 'EE' and 'WE'), and in a few mongooses and skunks [7]. The definitive demonstration of adaptive evolution in these cases clearly requires extensive experimental analysis in animals using some of these mutant viruses obtained by reverse genetics. However, our experimental results confirm that host shifts in RABV involved limited and unique sets of substitutions as observed *in natura* in bats [18, 29] and in non-flying mammals [7]. Further, our data support the concept of fortuitous ancestral pre-adaptation in RABV evolution that allows some RABV to more easily shift hosts into terrestrial mammals [4, 7], although this process is unidirectional as the dog-adapted rabies virus appears more prone to jump to fox than the reverse. Further, our study shows that if a virus is already genetically competent for a host shift prior to transmission rather than undergoing genetic adaptation in the new host [20, 29], the genetic diversity that could be linked to adaptation to a new host species is not present (even partially as shown by in-depth NGS data) in the donor host.

Although the control of rabies in dogs is the priority from a public health perspective [36], the control of rabies in other wildlife reservoir species is also of fundamental importance as these species may participate to the maintenance of rabies in dogs. Indeed, the repeated spill-over between dogs and other carnivore species has been recorded in many parts of the world [22, 23, 37–42]. Further high resolution assessment of the role of cross-species transmission and adaptation in the maintenance of RABV in a defined environment are clearly needed to identify the determinants of RABV host switching and to inform the long-term control of rabies.

## Material and methods

### Ethics statement

Animal handling and care were performed in accordance with the French Animal Protection Law (decree 2013–118) and with the Directive 2010/63/EU of the European Parliament and of the Council on the protection of animals used for scientific purposes and experiments.

All experiments in dogs and red foxes were performed at the Anses–Nancy laboratory for rabies and wildlife (France). This laboratory has all the expertise, the regulatory and the technical facilities to perform animal experiments under J. Barrat's personal approval #54–60 in the A2/A3 facilities located at Atton, France. This facility has been approved by veterinary services under C-54-4311 between 19 April 2011 and 18 April 2016, then renewed for 6 years as D-54-4311. On the 15th May 2012, the project, registered 15/05/12-1, was approved by the ethics committee on animal experimentation of ANSES, ENVA and Université Paris-Est. Dogs (*Canis lupus familiaris*) were purchased from the accredited supplier CEDS, 89130 Mézilles, France. Red foxes (*Vulpes vulpes*) were purchased from Norges Pelsdyralslag, Olso, Norway and Luova Research, Kannus, Finland.

All mice experiments were performed at the Anses–Nancy laboratory for rabies and wildlife (France) according to the project, registered 12–053 and approved by the ethics committee on animal experimentation of ANSES, ENVA and Université Paris-Est, agreed by the Ministry of Research since 2010 and registered 16.

#### Serial passage of RABV in dogs and red foxes

For each RABV intra- and inter-host combination, the peripheral (intra-temporal muscle) route of inoculation was used to mimic the natural transmission of rabies. The submaxillar salivary glands of each rabid animal were excised during necropsy and used for subsequent passages using the same route of inoculation and a similar dose. All inoculated animals were observed daily, and at least twice during the clinical phase. For ethical reasons, the end-point for humane euthanasia of animals was fixed to paralysis or severe self-occasioned wounds. All animals, dead or euthanized because they had reached the end-point, were necropsied. Brain tissues and salivary glands were collected, tested for RABV positivity and used to perform NGS. Samples were tested for RABV positivity by FAT and RTCIT [43] and the number of viral RNA copies present in the salivary glands were further controlled by RT-qPCR [44] (no quantification was performed on the brain tissues). The vDog and vFox RABV strains used for passages were 'Ariana2' from Tunisia [45, 46] (Genbank accession number: MK981888) and '91047FRA' collected from a rabid fox in France in 1991 [47] (Genbank accession number: KX148127), respectively. All strains were originally obtained from infected salivary glands and were never passaged in cell culture nor in other animal species. For each passage, doses of vDog and vFox inoculated to dogs and foxes were verified by mice intra-cerebral injection [48]. Doses of vDog and vFox inoculated to foxes were 2.2 (SD = 0.8) and 2.2 (SD = 0.4) mice intra-cerebral LD50 (MIC LD50), respectively. Doses of vDog and vFox inoculated to dogs were 2.1 MIC LD50 (SD = 0.9) and 1.7 MIC LD50 (SD = 0.7), respectively (S1 Table).

#### Primary brain cell cultures, virus infection and passages

Dog foetuses were obtained after 40 days of gestation by ovary-hysterectomy of a pregnant female. This surgical operation was performed by veterinarians of the unit Biology of Development and Reproduction of the Ecole Nationale Vétérinaire d'Alfort within the normal framework of their activities. As foetuses were collected before the last third of gestation, this study did not request the approbation of the national ethics committee on animal experimentation.

Fox brain tissues were collected from dead foetuses sampled from pregnant vixens that had been killed in the field by the Entente de Lutte Interdépartementale contre les Zoonoses within the framework of a survey on *Echinococcus sp*. in wild foxes following the French ministerial order NOR AGRG1238753A. Therefore, no fox was specifically killed for this study and this study does not fall into the legislation of animal experimentation according to the French decree 2013–118 and to the EU directive 2010/63/EU.

Brain cells were prepared from cortex of 3 dog foetus and 4 fox embryos (8 cm long without tail) by a mechanical dissociation and stored in liquid nitrogen. Upon use, neurons were directly plated on variable supports, Lab-Tek Chamber Slides to perform indirect immunofluorescence or culture plates for passages. Before seeding, supports were coated with 1.5μg/ml poly-ornithine (Sigma). Brain cells were maintained in Neurobasal medium (Invitrogen) supplemented with 0.1% penicillin-streptomycin (Gibco), 1% Glutamax (Life Technologies SAS), and 2% B-27 supplement (Life Technologies SAS) and seeded for the passages at 50000 cells per well in 12 well plates. 14 days after plating, virus infection was performed during 4 days at 37°C using vDog or vFox (same strains as *in vivo* passages) at a multiplicity of infection (MOI) of 1 when possible or with a lower MOI in the case of viruses with a lower titre (S2 Table). Infected brains cells were then used to produce a new stock of viruses for the following passage, viruses were titrated on Neuro 2A cells (ATCC CCL 131) at each passage. In parallel, infected cells were freezed-thawed and total RNA was extracted from cells in 300 μl to perform NGS.

### Indirect immunofluorescence analysis

Cells were fixed and permeabilized with 80% acetone at 4°C for 30 min followed by a blocking step with PBS 1X–10% SVF for 20 min at room temperature. Cells were washed 3 times in PBS 1X and primary antibodies were incubated 1 h at room temperature. To stain both neuronal and glial cells present in the culture, we used a mouse anti-MAP2 antibody (at a concentration of 1/1000) (MAB378, Millipore) and a rabbit anti-GFAP antibody (at a concentration of 1/1000) (AB5804, Millipore), respectively. Upon incubation with primary antibodies, cells were washed 3 times with PBS 1X and incubated with secondary goat anti-mouse antibody coupled to DyLight 549 (at a concentration of 1/1000) (072-04-18-03, KPL) or goat anti-rabbit antibody coupled to DyLight405 (072-08-15-06, KPL), for 1 h at room temperature. For controlling infection, we then used the Light Diagnostics Rabies DFA Reagent (at a concentration of 1/60) (Millipore) for 1 h at room temperature. 15 minutes before the end, TO-PRO-3 (ThermoFisher Scientific) was added to stain cell nuclei and 3 final washes were performed. Images were acquired using aa Zeiss Axioplan fluorescence microscope equipped with a Zeiss ApoTome system (obj.10X).

### RNA extraction and next-generation sequencing

Total RNA (final volume 50 μl) was extracted from brain or salivary gland samples or after brain cells passages using Trizol (Ambion) according to the manufacturer's instructions. 5 μl of RNA were then reverse transcribed using Superscript III reverse transcriptase with random hexamers (Invitrogen) according to manufacturer's instructions. Although non-standardized input concentrations of viral RNA have been used for PCR amplification before NGS, the number of RNA copies/ul of cDNA determined from salivary glands and the virus titres of samples obtained for the *in vitro* passages indicate that more than 1000 virus RNA copies were used (S1 and S2 Tables). No quantification was performed on the brain samples. The complete viral genome (excluding the 3' and 5' extremities, corresponding to the leader and the trailer regions, respectively) was amplified with 6 overlapping PCR fragments by using the Phusion polymerase (ThermoFisher) as described previously [7]. After electrophoresis, each PCR fragment was independently purified using the NucleoSpin Gel and PCR clean-up kit (Macherey-Nagel) and quantified using Picogreen dsDNA quantification kit (Invitrogen). For each sample, all six PCR fragments were pooled with equimolar proportions to obtain 500 ng of dsDNA. For the preparation of libraries and next-generation sequencing, dsDNA was fragmented by ultrasound with Bioruptor (Diagenode), libraries were prepared using NEXTflex PCR-Free DNA-Seq kit (Bioo Scientific), and then sequenced using a 2 x 300 nucleotides paired-end strategy on the Illumina MiSeq platform.

### Genome sequence analyses

All sequencing reads were pre-processed to remove low-quality or artefactual bases. fqCleaner v.0.5.0, a mini workflow implemented in Galaxy (doi:10.1093/nar/gkw343, doi:10.7490/f1000research.1114334.1, https://galaxy.pasteur.fr/) was used for preprocessing fastq files, it includes quality trimming, duplicate and artefacts filters. Sequences of the PCR primers (S3 Table) were removed from all analyses. However, because there were mismatches between the primers and the RABV genome sequences it is possible that these mismatches have had a minor impact on our estimates of SNV frequencies [49], in a manner that is not easy to quantify. Reads with length of less than half of the original read (300bp) or those containing >20% of bp with a Phred score of <25 were discarded. The filtered reads were then mapped with 0.98 similarity to the closest RABV sequence (GenBank references for 91047FRA: KX148127 and GenBank reference of an isolate (RV2627) from Morocco related to the Ariana 2 strain (KF155001.1)) using the CLC Genomics Assembly Cell (v4.0) implemented in Galaxy. To investigate minority (i.e. sub-consensus) variation, an arbitrary cut-off frequency of 2% was used to consider variants as significantly different from artefactually introduced reverse-transcription, PCR and sequencing errors. The detection of these Single Nucleotide Variations (SNVs) was performed using the clc_find_variations tool of CLC.

Finally, to help analyze selection pressures, we determined the ratio of non-synonymous (*d*_*N*_) to synonymous (*d*_*S*_) nucleotide substitutions per site based on the consensus level sequence data using the Single Likelihood Ancestor Counting (SLAC) method available in the Datamonkey web server of the HyPhy package [50, 51] as previously described [7]. This analysis utilized the two reference sequences of the challenge viruses.

## Supporting information

S1 Fig Coverage of the rabies genome. A schematic representation of the RABV genome is shown at the top of the figure. The number of mapped reads are per position projected along the RABV genomic position. Peaks are related to PCR fragments overlapping areas. (A) *In vitro* experiments. (B) *In vivo* experiments. The different colors correspond to different samples (different passages in animals or in cells).
(PDF)

S2 Fig Average number of total SNVS distributed throughout RABV genome. SNVs are plotted as the percentage per nucleotide per region (N, P, M, G and L genes and the non-coding (NC) regions) and are representative of the average number of mutations per region and per animal for in vivo passages (in black) or per cell culture for in vitro passages (in grey). Error bars correspond to standard deviations.
(PDF)

S3 Fig Evolution of genetic diversity during in vivo experimental passages. The genetic diversity of each experiment was determined at each passage by calculating the mean substitution rate per animal ± SD.
(PDF)

S1 Table Doses of inoculum (MIC LD50) used to infect the following in vivo passage. Quantification of viral RNA copies (RNA copies /μl of cDNA) present in the salivary glands used for PCR amplification and NGS. Numbers of passages (P1 to P4) refer to Fig 1. X: undetectable viral loads. ND: not determined.
(PDF)

S2 Table Doses of inoculum (UFF/ML) used to infect the following in vitro passage. Numbers of passages (P1 to P5) refer to Fig 1. X: undetectable viral loads.
(PDF)

S3 Table List of primers used in this study. (PDF)
