# Supplementary material for: Comparison of intra- and inter-host genetic diversity in rabies virus during experimental cross-species transmission
Source: PLoS Pathog. 2019 Jun 20;15(6):e1007799. doi: 10.1371/journal.ppat.1007799 (PMC6615636; doi:10.1371/journal.ppat.1007799)

Figure S1. Coverage of the rabies genome.

A schematic representation of the RABV genome is shown at the top of the figure. The average number of mapped reads are per position projected along the RABV genomic position. Peaks are related to PCR fragments overlapping areas. (A) *In vitro* experiments. (B) *In vivo* experiments. The different colors correspond to different samples (different passages in animals or in cells)

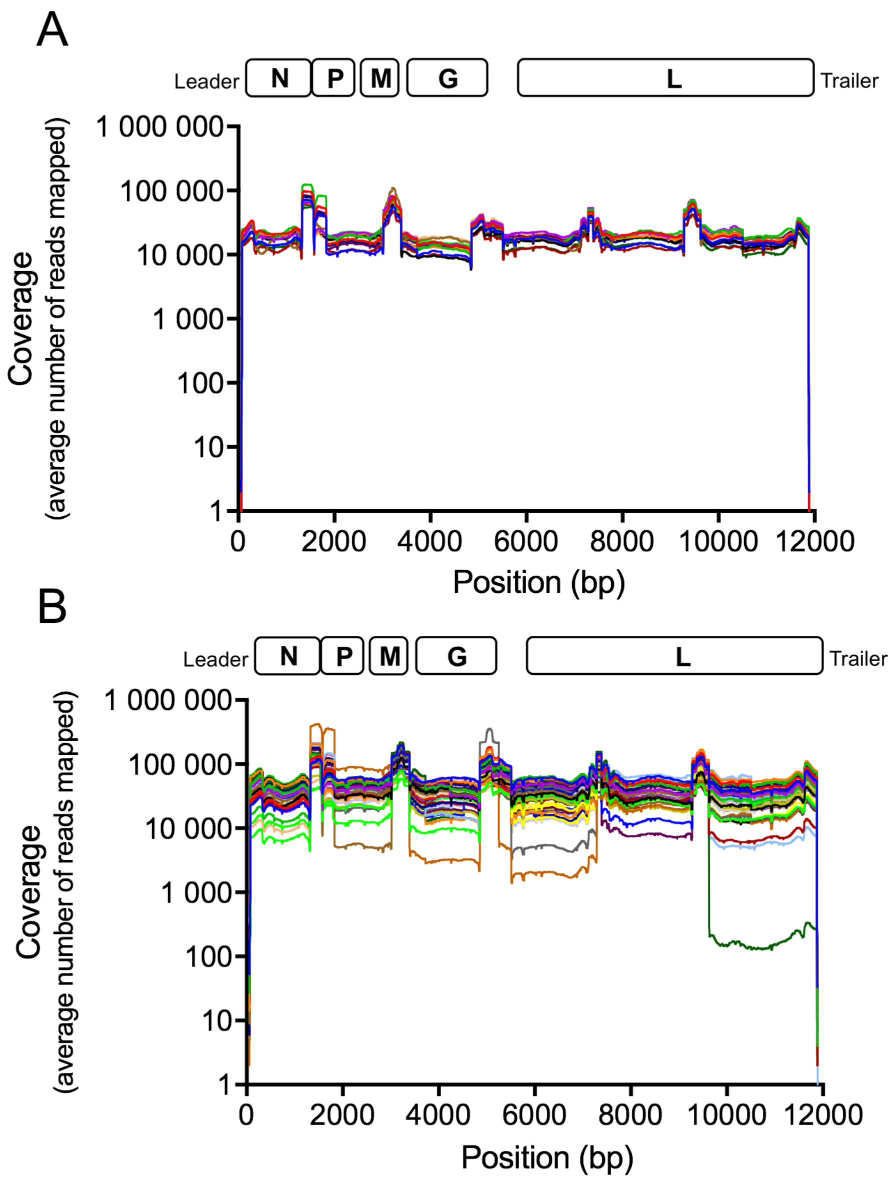

Supplement: S1 Fig — A schematic representation of the RABV genome is shown at the top of the figure. The number of mapped reads are per position projected along the RABV genomic position. Peaks are related to PCR fragments overlapping areas. (A) In vitro experiments. (B) In vivo experiments. The different colors correspond to different samples (different passages in animals or in cells). (PDF) [file ppat.1007799.s001.pdf]
