# Supplementary material for: Comparison of intra- and inter-host genetic diversity in rabies virus during experimental cross-species transmission
Source: PLoS Pathog. 2019 Jun 20;15(6):e1007799. doi: 10.1371/journal.ppat.1007799 (PMC6615636; doi:10.1371/journal.ppat.1007799)

Figure S2. Average number of total SNVs distributed throughout RABV genome.
SNVs are plotted as the percentage per nucleotide per region (N, P, M, G and L genes and the non-coding (NC) regions) and are representative of the average number of mutations per region and per animal for *in vivo* passages (in black) or per cell culture for *in vitro* passages (in grey). Error bars correspond to standard deviations.

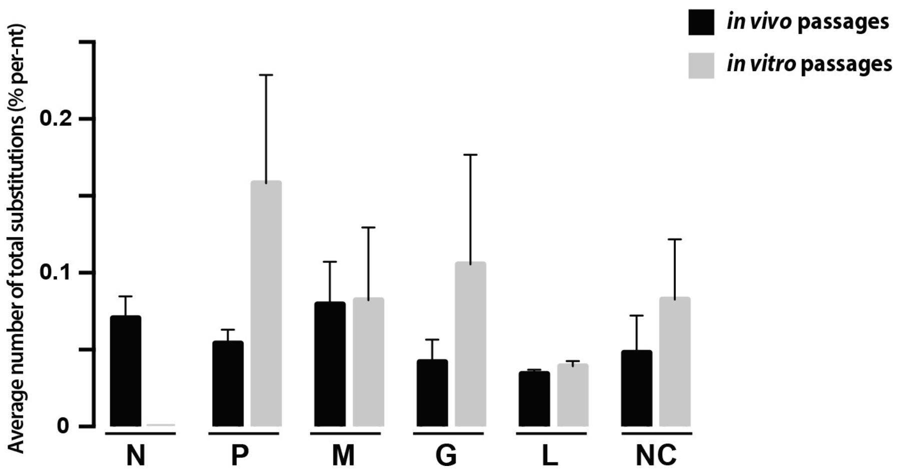

Supplement: S2 Fig — SNVs are plotted as the percentage per nucleotide per region (N, P, M, G and L genes and the non-coding (NC) regions) and are representative of the average number of mutations per region and per animal for in vivo passages (in black) or per cell culture for in vitro passages (in grey). Error bars correspond to standard deviations. (PDF) [file ppat.1007799.s002.pdf]
