# Supplementary material for: Comparison of intra- and inter-host genetic diversity in rabies virus during experimental cross-species transmission
Source: PLoS Pathog. 2019 Jun 20;15(6):e1007799. doi: 10.1371/journal.ppat.1007799 (PMC6615636; doi:10.1371/journal.ppat.1007799)

Figure S3. Evolution of genetic diversity during *in vivo* experimental passages.

The genetic diversity of each experiment was determined at each passage by calculating the mean substitution rate per animal ± SD.

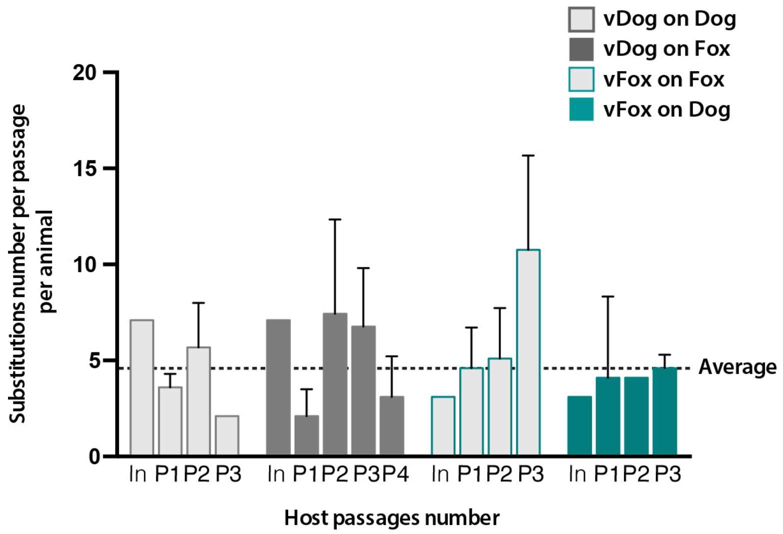

Supplement: S3 Fig — The genetic diversity of each experiment was determined at each passage by calculating the mean substitution rate per animal ± SD. (PDF) [file ppat.1007799.s003.pdf]
