# Supplementary material for: Comparison of intra- and inter-host genetic diversity in rabies virus during experimental cross-species transmission
Source: PLoS Pathog. 2019 Jun 20;15(6):e1007799. doi: 10.1371/journal.ppat.1007799 (PMC6615636; doi:10.1371/journal.ppat.1007799)
Supplement: S1 Table — Quantification of viral RNA copies (RNA copies /μl of cDNA) present in the salivary glands used for PCR amplification and NGS. Numbers of passages (P1 to P4) refer to Fig 1. X: undetectable viral loads. ND: not determined. (PDF) [file ppat.1007799.s004.pdf]

Table S1 : Doses of inoculum (MIC LD50) used to infect the following *in vivo* passage. Quantification of viral RNA copies (RNA copies /µl of cDNA) present in the salivary glands used for PCR amplification and NGS. Numbers of passages (P1 to P4) refer to Figure 1. X: undetectable viral loads. ND: not determined.

Virus	Host species	Replicate	P0	P1	P2	P3	P4
**vDog**	**Dog**	1	2.6	2.2 (5.3)	X (3.7)		
		2	2.6	0.4 (5.3)	(4.2)		
		3	2.6	0.4 (5.3)	0.1 (4.5)	(4.8)	
		4	1.6	0.9 (5.7)	(3.1)		
		5	1.6	0.9 (5.7)	1.7 (3.6)	(2.6)	
	**Fox**	1	3.4	2.3 (6.3)	1.3 (6.2)	(6.1)	
		2	2.1	2.5 (5.8)	3.3 (4.6)	1.3 (6.0)	(ND)
		3	2.1	2.5 (5.8)	1.7 (5.7)	1.4 (5.6)	(6.2)
**vFox**	**Fox**	1	1.6	2.7 (7.5)	2.6 (6.7)	(6.5)	
		2	1.6	2.1 (6.7)	2.4 (ND)	(6.5)	
		3	1.6	2.1 (6.7)	2.6 (7.2)	(7.4)	
	**Dog**	1	2.5	1.8 (5.3)	0.5 (4.6)	(4.3)	
		2	2.5	1.8 (5.3)	1.5 (4.6)	(4.0)	
		3	2.5	X			
		4	1.5	(5.0)			
		5	1.5	X			
