# Supplementary material for: Comparison of intra- and inter-host genetic diversity in rabies virus during experimental cross-species transmission
Source: PLoS Pathog. 2019 Jun 20;15(6):e1007799. doi: 10.1371/journal.ppat.1007799 (PMC6615636; doi:10.1371/journal.ppat.1007799)
Supplement: S2 Table — Numbers of passages (P1 to P5) refer to Fig 1. X: undetectable viral loads. (PDF) [file ppat.1007799.s005.pdf]

Table S2 : Doses of inoculum (UFF/ml) used to infect the following *in vitro* passage.
Numbers of passages (P1 to P5) refer to Figure 1. X: undetectable viral loads.

Virus	Cell species	Replicate	P0	P1	P2	P3	P4	P5
**vDog**	**Dog**	1	6.1	3.6	4.6	5.1	5.4	5.5
		2	6.1	3.8	4	4.8	5.3	5.3
		3	6.1	3.8	4.5	5.3	5.4	5.7
	**Fox**	1	6.1	4 .6	4	4.4	5.3	5.2
		2	6.1	4.7	3.6	4.7	5.7	5.4
		3	6.1	4	3.2	3.8	5.2	X
**vFox**	**Fox**	1	6.2	3.7	3.1	3.8	4.4	4.6
		2	6.2	3.7	2.3	3.1	3.9	4.4
		3	6.2	2.6	2.7	X		
		4	6.2	2.8	X			
	**Dog**	1	6.2	3.2	X			
		2	6.2	3.2	2.2	X		
		3	6.2	3.3	X			
		4	6.2	3.3	X			
