# Supplementary material for: Comparison of intra- and inter-host genetic diversity in rabies virus during experimental cross-species transmission
Source: PLoS Pathog. 2019 Jun 20;15(6):e1007799. doi: 10.1371/journal.ppat.1007799 (PMC6615636; doi:10.1371/journal.ppat.1007799)
Supplement: S3 Table — (PDF) [file ppat.1007799.s006.pdf]

Table S3: List of primers used in this study.

Primer Name	Primer sequence (5' to 3')[a]
N127	F: ATGTAACACCTCTACAATGG
N8m	R: CAGTCTCYTCNGCCATCTC
N1304-S3	F: AAYGGAGGTCGACTVAARAGATC
G3393-AS3	R: CADGGRCCNAGYTTGTCTGGTAT
M220	F: TGGTGTATCAACATGRAYTC
L1	R: GAGTTNAGRTTGTARTCAGAG
G4836-S3	F: GGRARRGTYATATCTTCNTGGGA
L7386-AS3	R: CTRTCBGARTARTADAYCCANGACTT
PVO8	R: GGTCTGATCTRTCWGARYAATA
Taq3long	F: ATGAGAAGTGGAAYAAYCATCA
L9633-AS3	R: TGCYRTATATGTTGACAGG
L9267-S3	F: ATGTTYCAGCCNTTGATGCT
L9129-S3	R: TCNGCCTTGCAYAGGTTCAA
L11872-AS3	R: AAAYAATCAARCARHCAGAGG

[a] F: Forward; R: reverse
